# Integrating of Radiomics and Single-cell Transcriptomics Uncovers the Crucial Role of γδ T Cells in Lung Adenocarcinoma

**DOI:** 10.7150/jca.105586

**Published:** 2025-01-06

**Authors:** Ziyi Chen, Changqing Yang, Jiajing Wang, Baichuan Wang, Long He, Zhaoyi Liu, Yingxi Li, Tingting Qin, Peng Chen

**Affiliations:** 1 Tianjin Medical University Cancer Institute and Hospital, National Clinical Research Center for Cancer; Key Laboratory of Cancer Prevention and Therapy, Tianjin, China.; 2 Tianjin's Clinical Research Center for Cancer, Tianjin, China.; 3 Department of Thoracic Oncology, Tianjin Lung Cancer Center, Tianjin Cancer Institute & Hospital, Tianjin Medical University, Tianjin, 300060, PR China; 4 Respiratory Department, Tianjin Medical University General Hospital, Tianjin 300052, China.; 5 Anhui Chest Hospital, Anhui Medical University Clinical College of Chest, Hefei, Anhui Province, 23002, China.; 6 Department of General Surgery, Tianjin Medical University General Hospital, Tianjin Key Laboratory of Precise Vascular Reconstruction and Organ Function Repair, Tianjin General Surgery Institute, Tianjin, 300052, China.; 7 Immunology Department, Key Laboratory of Immune Microenvironment and Disease (Ministry of Education), Tianjin Medical University, Tianjin, 300070, China.

**Keywords:** gamma-delta T cells, lung adenocarcinoma, tumor microenvironment, immunotherapy, radiomics

## Abstract

Gamma-delta (γδ) T cells are a crucial component of the tumor immune microenvironment which are considered a promising potential therapeutic strategy and target. Increasing evidence suggests that these unique immune cells play significant roles across various cancers. However, γδ T cells are often regarded as having dual roles in tumors, and their influence on lung adenocarcinoma (LUAD) remains controversial. In this research, we employed a wild-ranging approach using multi-omics data to investigate the function of γδ T cells in LUAD. The abundance of γδ T cell infiltration is linked to a positive prognosis in lung adenocarcinoma. The tumor-inhibiting role of γδ T cells was played through intrinsic lineage evolution, acquiring cytotoxic functions and engaging in signal transduction with antigen-presenting cells. Furthermore, patients with higher γδ T cells infiltration abundance might be more favorable for immunotherapy. Lastly, we established a predictive model using CT images based on radiomics, providing a non-invasive strategy to assess γδ T cells infiltration in LUAD patients. These findings offer new insights and perspectives the personalized therapies of γδ T cells.

## Introduction

γδ T cells represent an emerging field in tumor immunology, and their effects on the tumor microenvironment and therapeutic applications remain unclear compared to CD8+ and CD4+ T cells. In recent years, research related to γδ T cells has attracted more and more attention in the academic community. γδ T cells represent a distinct subset of infiltrating lymphocytes in the tumor microenvironment, possessing important and distinct functions and characteristics. Compared to the typical αβ T cells, γδ T cells possess a unique T cell receptor on their surface, which is constituted of both a γ chain and a δ chain^1^. Meanwhile, γδ T cells have the ability to detect the early changes when normal cells transform into tumor cells and they can sense tumors even in the presence of low mutational burden, exhibiting a certain degree of sensitivity and a rapid response^1^. Notably, γδ T cells possess multifaceted immune characteristics, as they not only directly kill tumor cells but also regulate immune function indirectly through the secretion of cytokines, such as IFN-α and TNF-γ thereby activating other immune cells^2^. Currently, conventional immunotherapies primarily rely on alpha-beta T cells, which are strictly dependent on MHC molecules, substantially reducing the therapeutic effectiveness and usability in different situations^3^. In contrast, one of the key advantages of γδ T cells is the MHC independence, allowing for allogeneic transplantation and they have been reported to be correlated with survival rates in multiple cancers, showing promising antitumor potential and application prospects^4^. The γδ T cells possess unique clinical translational value and hold significant promise in cancer therapy. Nevertheless, there is still evidence indicating that γδ T cells may promote tumorigenesis across various cancers. To give an example, Reis BS *et al.* discovered that γδ T cells could exhibit effects of promoting tumor in colorectal cancers (CRCs)^5^. Similarly, there were reports that γδ T cells may make a positive contribution in tumor growth in ovarian cancer^6^. Consequently, it is crucial to perform a detailed investigation of the function of γδ T cells in particular cancer types.

Lung cancer has consistently ranked among the most prevalent and fatal malignancies worldwide^7^. Characterized by high incidence and mortality rates, lung cancer imposes a substantial burden on global healthcare systems^8^, ^9^. More than 80% of all lung cancers are classified under the NSCLC, of which lung adenocarcinoma (LUAD) consists of the majority subtype^10^, ^11^. The molecular heterogeneity within and between tumors has long posed a major obstacle in treating patients with lung adenocarcinoma^12^, ^13^. Furthermore, issues such as drug resistance and metastasis have led to unsatisfactory prognoses for lung adenocarcinoma^14^. Thus, it is essential to discover new therapeutic targets and risk stratification strategies with clinical translational significance in lung adenocarcinoma against these challenges, ultimately benefiting a greater number of patients.

Single-cell RNA sequencing technology has made significant progress in recent years, providing a novel research perspective for further exploration of the tumor microenvironment^15^. The sc-RNA technology offers unique advantages in revealing the intra-tumoral heterogeneity and interactions among different cellular components^16^. Radiomics presents a clinical application method for disease assessment that is characterized by non-invasiveness, reproducibility and cost-effectiveness^17^. The integrated application of various technologies could better assist researchers in analyzing the properties of the TME.

In the UK project TRACERx of lung cancer, the researchers evaluated lung tumor tissues and adjacent cancerous samples from 25 individuals with NSCLC and found that γδ T cells were indeed infiltrated in the tumor^18^. Nevertheless, the biological function of γδ T cells in lung adenocarcinoma still remains controversial, and their specific function within this cancer type have seldom been explored through multi-omics profiling methods. Based on this fact, we employed multi-omics data, including sc-RNA sequencing data, bulk transcriptomic data, somatic mutation data, immune response data, and CT imaging radiomics data, to investigate the potential duty of γδ T cells in the TME of LUAD.

We discovered that γδ T cells in the TME of LUAD were associated with both favorable prognosis and downregulation of malignant pathways, and they might possess an intrinsic developmental trend that further enhances their direct cytotoxic function and immune regulatory roles. Notably, somatic mutations in genes such as Lipoprotein-A (*LPA*), Dynein Axonemal Heavy Chain 8 (*DNAH8*), and Tenascin N (*TNN*) may serve as driver genes that induce these differences. Furthermore, γδ T cells were demonstrated to have closely connection with immune therapy. Additionally, we established a clinical application model to assess the infiltration levels of γδ T cells through CT imaging. Overall, the further research to investigate the prognostic value of γδ T cells in tumors and their potential as a key therapeutic strategy were crucial for patients with lung adenocarcinoma.

## Materials and Methods

### Data collection and preliminary processing

The sc-RNA profile GSE223923 containing four samples from different lung adenocarcinoma patients utilized in the research was obtained from the Gene Expression Omnibus Series^19^. Bulk RNA transcriptome data, clinical data, and single nucleotide variant (SNV) data were gathered from the LUAD cohort of The Cancer Genome Atlas (TCGA, https://portal.gdc.cancer.gov/) via the 'TCGAbiolinks' package^20^. CT image data for radiomics were collected from The Cancer Imaging Archive (TCIA, https://dev.cancerimagingarchive.net/)^21^. Single-cell RNA profiles were analyzed applying the Seurat (version 4.3.0) R package. Regular quality control (The QC standards can be found in detail in Supplementary Figure 1) was conducted on each scRNA sequencing data required and the software package DoubletFinder (version 2.0.3)^22^ was employed to detect and filter potential cell doublets. Harmony (version 1.2.0)^23^ was employed to eliminate batch effects and integrate information across different samples which could be found in Supplementary Figure 2. Sctransform (version 0.3.5)^24^ was used for data normalization and removal of confounding sources of variation, such as mitochondrial interference percentage. Clustree (version 0.5.1)^25^ was used to visualize the resolution for further selection.

### Downstream analysis of single-cell RNA transcriptome data

The annotated biological markers for different cell populations were derived from the online database CellMarker 2.0^26^ and previously published studies. Intrinsic spectral evolution and developmental trend of γδ T were analyzed using monocle (version 2.26.0)^27^ with 'DDRTree' being the default dimensionality reduction method and the max component was set to 2. Cell-to-cell communication analysis was performed with CellChat (version 1.6.1)^28^ and call CellChatDB.human database for analysis. To estimate the infiltration abundance of γδ T cells, we employed the CIBERSORTx platform^29^ which is an analytical tool designed to deconvolute immune cell composition based on RNA-seq data. CIBERSORTx deconvolution algorithm could utilize a reference expression profile to calculate the relative fraction of γδ T cells in absolute mode based on bulk sequence data.

### Gene enrichment analysis

We utilized the limma package (version 3.58.1)^30^ to identify the differential expression genes between the different γδ T cell infiltration abundance groups, defining genes with a p < 0.05 as statistically significant and ranking them based on the log fold change (logFC) of their expression levels. Gene sets data were obtained from the Gene Set Enrichment Analysis (GSEA, https://www.gsea-msigdb.org/gsea/index.jsp) database^31^. The enrichment analysis results were visualized with the 'clusterProfiler' package (version 4.12.2)^32^ and the 'GseaVis' package (version 0.0.5).

### Somatic mutation landscape and survival analysis

The 'TCGAbiolinks' package was employed to retrieve the required single nucleotide variant (SNV) mutation data from the Cancer Genome Atlas (TCGA) database as mentioned above. Comprehensive analysis and visualization of masked somatic mutation profiles were conducted with the 'maftools'^33^ package (version 2.20.0). Fisher's exact test was employed to recognize significant gene pairs between different groups. Survival prognostic analyses were achieved with the 'survminer' package (version 0.4.9) and p < 0.05 was defined as statistical significance.

### Computerized estimation of treatment response to ICIs

The potential response of immune checkpoint inhibitors (ICIs) was assessed by TIDE algorithm. TIDE^34^ was a tool based on tumor immune escape mechanism that integrates immune dysfunction and immune rejection features. To make the results as reliable as possible, the response scores of the different γδ T cell infiltration abundance groups to immunotherapy were predicted using the EaSIeR package (version 1.10.0)^35^ in combination with the responder data obtained from TIDE and the tumor mutation burden (TMB) data. The cell signature of the γδ T cells population was calculated via the ssGSEA algorithm of the 'GSVA' package (version 1.50.5)^36^ by top30-markers. The relationships between immunotherapeutic targets and signature score were investigated with Pearson correlation analysis.

### Image segmentation, feature extraction and modelling for radiomics

The CT image data of 29 lung adenocarcinoma patients from The Cancer Imaging Archive (TCIA) with corresponding bulk transcriptome data was selected. The workflow was based on 3D Slicer software (version 5.6.2)^37^ and its plugins. The process of region of interest (ROI) delineation and feature extraction adhered to the guidelines of The Image Biomarker Standardization Initiative^38^. Tumor regions were segmented by radiologists with over five years of experience, followed by review and confirmation by a senior clinician with over 20 years of experience. In cases of disagreement, an additional senior clinician was consulted for review. The Radiomics plugin was used to extract 851 radiomic features after the tumor regions were segmented layer by layer and reconstructed into three-dimensional images. To ensure uniform spacing resolution, all CT images were resampled into the same value during preprocessing. All extracted radiomic features underwent Z-score normalization. The correlation between the radiomics score and γδ T cell infiltration abundance was conducted with Pearson correlation analysis. The 'glmnet' package (version 4.1-8)^39^ was used with cross-validation to determine the best λ value, and a least absolute shrinkage and selection operator (LASSO) regression model was developed to predict the infiltration abundance scores of γδ T cells. Finally, the cohort was split into training and validation cohorts in a 2:1 ratio, and Pearson correlation was used to assess the model's performance.

### Statistics

Relevant methods of statistical analyses are described above, and all statistical methods were analyzed by R (v 4.3.3).

## Results

### Identification of γδ T cells in sc-RNA profile

**Figure 1:**
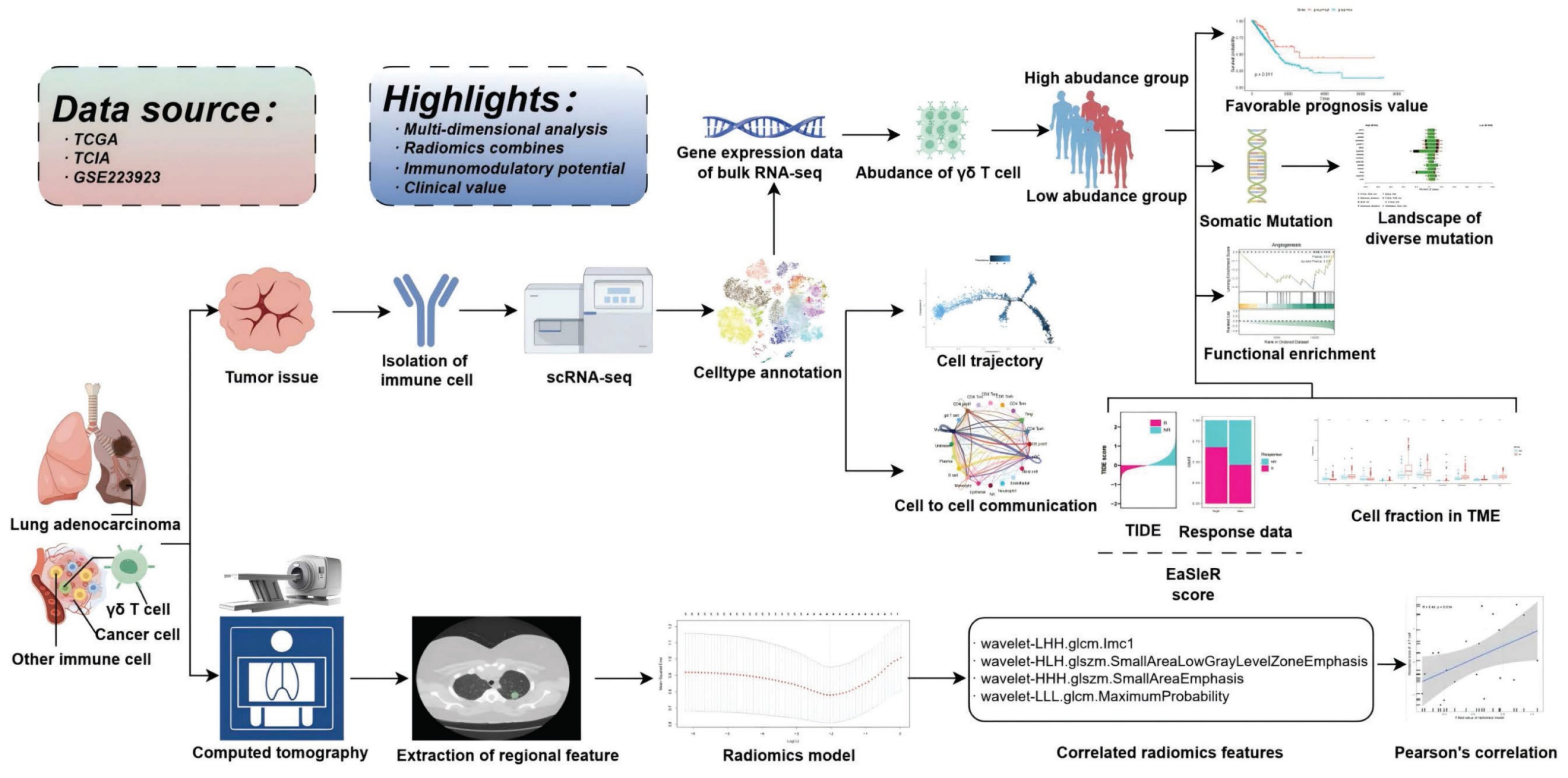
Schematic flowchart of the study.

**Figure 2:**
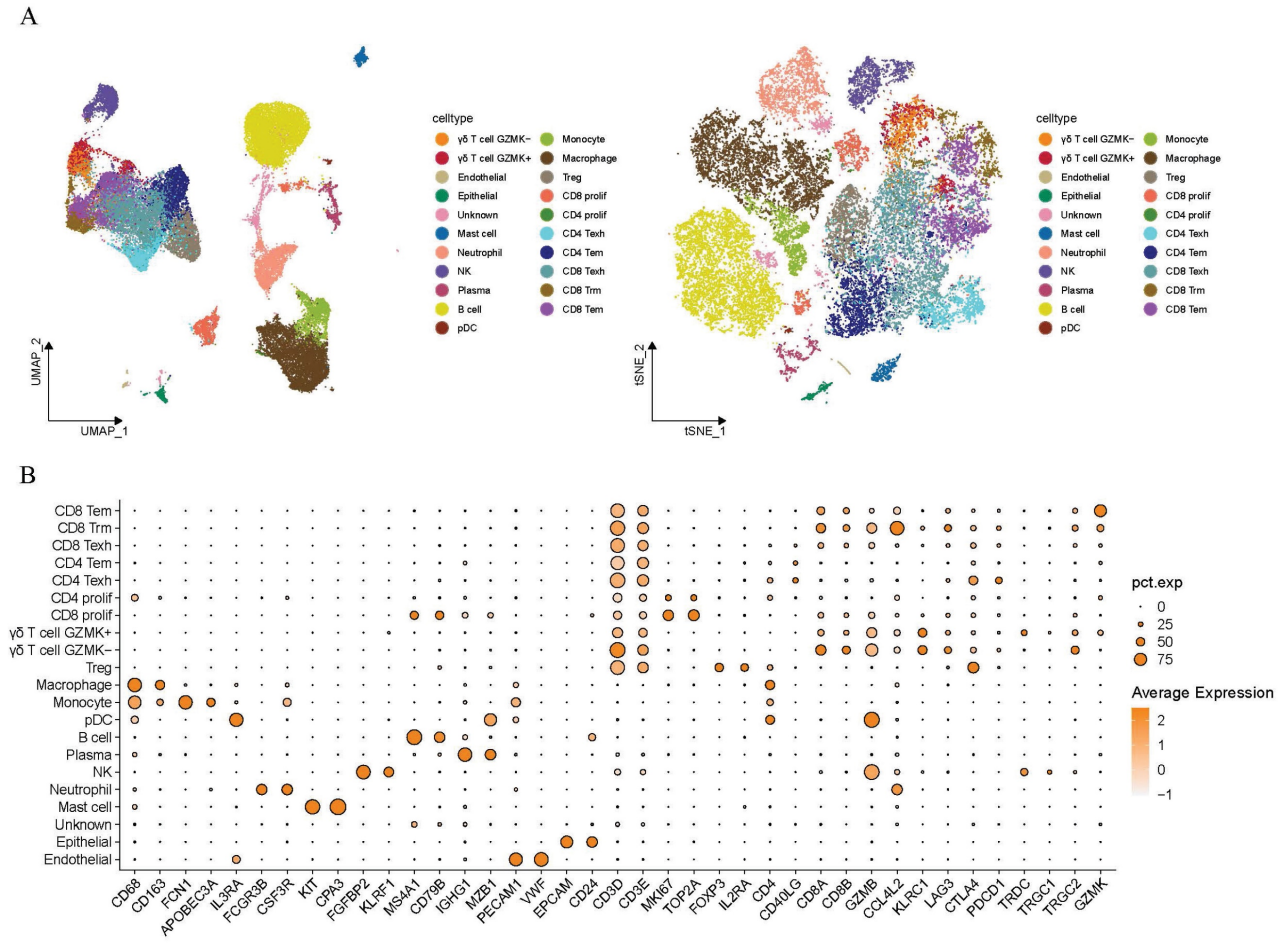
** Tracking and annotating γδ T cells from single-cell transcriptome data of lung adenocarcinoma.** (A) Uniform Manifold Approximation and Projection (UMAP)based (left) and t-Distributed Stochastic Neighbor Embedding (t-SNE)-based (right) dimensionality reduction map. The cell cluster annotations are marked with different colors. (B) The expression levels of cell marker genes in different cell cluster annotations are demonstrated by dotplot. The Y-axis represents the identity of cell cluster, the X-axis indicates the names of marker genes. The color of the dots represents the average expression level of the gene in the cell clusters. The size of the dots represents the proportion of gene expression in the corresponding cell clusters. Tem, effector memory T cells; Trm, tissue-resident memory T cells; Texh, exhausted T cells; Prolif, proliferating markers; Treg, regulatory T cells; pDC, plasmacytoid dendritic cell; NK, natural killer cell; Endothelial, endothelial cells; Epithelial, epithelial cells.

As illustrated in Figure 1, this study intended to probe the existence of γδT cells in the TME of LUAD and their potential value in clinical application. The sc-RNA sequencing data from GSE223923 of LUAD-infiltrating immune cells isolated using PE-Cy7-conjugated CD45 antibody fluorescent dye from lung adenocarcinoma patients was selected for analysis to more precisely identify γδ T cells. We then conducted an initial screening of the single-cell RNA profiles of the selected γδT cells. After removing potential doublets, the data quality and cell purity were deemed satisfactory. Following sequential batch effect removal and normalization processes, the cells were preliminarily classified into myeloid cells, endothelial cells, B cells, NK cells, plasma cells, epithelial cells and T cells. Further analysis divided the myeloid cells into neutrophils, monocytes, macrophages, pDCs, and mast cells. T cells were then subdivided into various subtypes, with a total of 1,818 γδ T cells identified for subsequent analyses. UMAP and t-SNE methods were employed for dimensionality reduction and visualization (Figure 2A). Specifically, the annotation of γδ T cells was further defined based on the expression levels of biological markers like *TRDC*, *TRGC1*, and *TRGC2*, with cell type annotation markers presented in Figure 2B.

### γδ T cells' cytotoxic and immunoregulatory roles via binary lineage evolution in the lung adenocarcinoma TME

**Figure 3:**
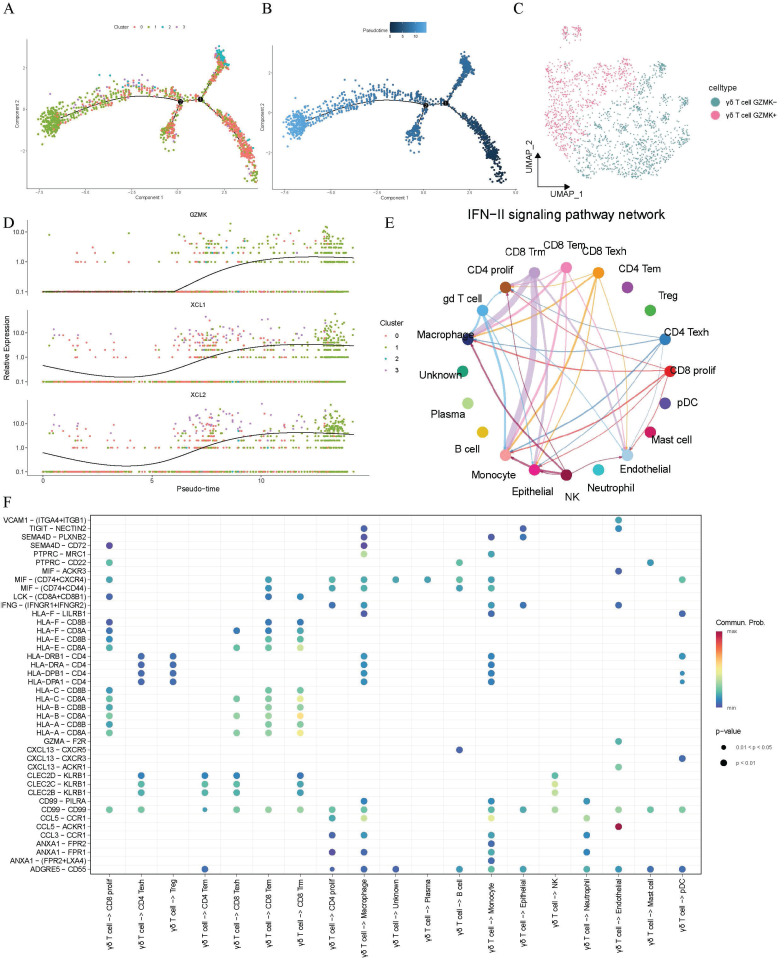
** Dynamic developmental transition of γδ T cells and their interactions with other cells components in the tumor microenvironment of lung adenocarcinoma.** (A-B) Pseudotime trajectory analysis showing the dynamic developmental transition of γδ T cells. Cells are colored by clusters (A) and pseudotime (B), illustrating distinct cellular states and transitions along the developmental trajectory. (C) The UMAP plot illustrates the manually defined dichotomous states of γδ T cells. (D) Dynamic gene expression of the genes of interest along the cellular trajectory developmental timeline. (E) Circular plot displaying the communication of the INF-γ signaling pathway between different cell components. (F) Dot plot illustrating the interactions of γδ T cells to other cells.

To further elucidate the distribution and trajectory of γδ T cells within the lung adenocarcinoma TME, pseudo-time analysis was conducted on the γδ T cells and we discovered an inherent lineage evolution process. These cells exhibited a unique developmental and evolutionary trend (Figure 3A-B). Based on their temporal progression, we found that γδT cells could be divided into two distinct bioactive states. After correcting for batch effects, the cells could be artificially classified into GZMK- and *GZMK+* γδT cells, representing different developmental maturity and activity states, based on the changes in the expression of the cytotoxic marker gene *GZMK* (Figure 3C). Along the trajectory of cellular evolution in pseudo-time, the cells dynamically transitioned from *GZMK-* to *GZMK+*, with the expression of the lymphocyte chemokine-encoding genes *XCL1* (lymphotactin-1) and *XCL2* (lymphotactin-2) progressively increasing over time (Figure 3D). Thus, γδ T cells demonstrated an inherent lineage development process that enhanced the immune functions within the lung adenocarcinoma TME. These cells exerted direct cytotoxic effects through the granzyme pathway and recruited additional immune cells via the lymphotactin pathway, thereby amplifying the anti-tumor immune response and modulating immune functions.

### Intercellular communication between γδ T cells and other cellular components in the TME of lung adenocarcinoma

Given the cell functions of γδT cells as a unique subtype of T cells, we had sufficient reasons to hypothesize that they might engage in potential intercellular communication with other cellular components in the tumor microenvironment, and that such interactions could be crucial for shaping the TME. Therefore, we conducted an in-depth investigation of the interaction network between γδ T cells and other cells. We observed IFN-γ signaling transmission between γδ T cells and macrophages as well as monocytes, which suggested their active role in antigen presentation. Similarly, CD8+ tissue-resident memory T cells exhibited comparable signaling towards macrophages and monocytes, indicating a potential synergistic effect between CD8+ Trm cells and γδ T cells in simultaneously activating these two cell types (Figure 3E). Furthermore, researchers found that γδ T cells extensively transmitted signals to CD8+ T cells through human leukocyte antigen class I molecule pathways, thereby triggering cytotoxic responses as illustrated in Figure 3F. γδ T cells also transmitted signals through *HLA* class II molecules to professional antigen-presenting cells (e.g., pDCs, monocytes, and macrophages) via pathways such as* HLA-DRB1-CD4*. This suggested that γδT cells may enhance tumor antigen presentation through these interactions, indirectly boosting the anti-tumor ability of CD4+ T cells. Notably, the *CCL5-ACKR1* pathway was significantly enriched in interactions from γδT cells to endothelial cells. These findings indicated that in addition to their direct cytotoxic effects, the indirect immunoregulatory functions of γδT cells were also an essential component.

### Infiltration of γδ T cells suggests favorable clinical prognosis

**Figure 4:**
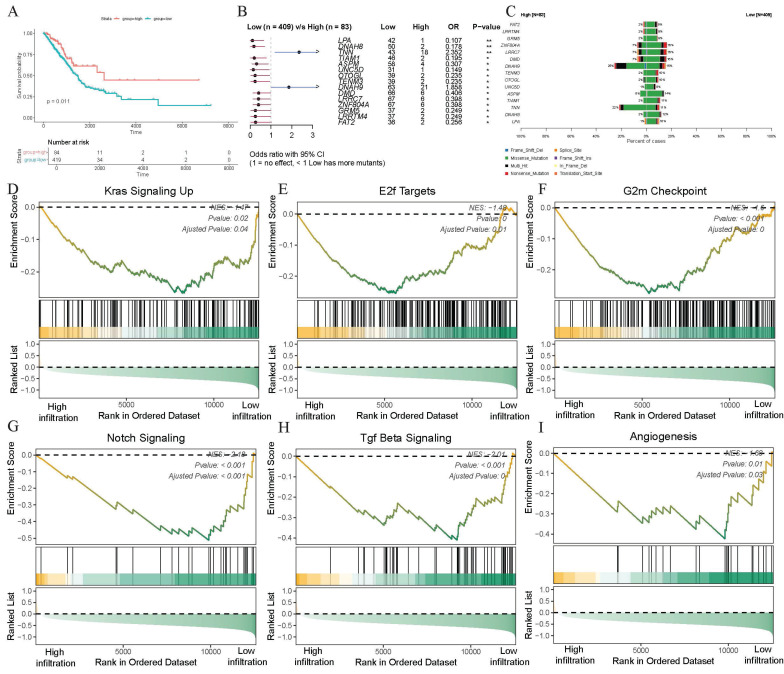
** Increased infiltration of γδ T cells is associated with better survival, somatic mutations, and decreased malignant tumor phenotypes.** (A) Kaplan-Meier curves describing overall survival (OS) in lung adenocarcinoma patients with different infiltration levels of γδ T cell infiltration. (B) Forest plot showing statistically significant differences in somatic single nucleotide variants (SNVs) between patients with high and low γδ T cell infiltration. OR > 1 indicates a higher frequency of SNVs in the high γδ T cell infiltration group, OR < 1 indicates a higher frequency in the low infiltration group, and OR = 1 indicates similar SNV frequencies between the two groups. (C) Bar chart displaying the mutation frequencies in two groups. (D-I) Significant enrichment of representative tumor malignancy pathways in patients with low γδ T cell infiltration based on hallmark gene set (D, *KRAS* signaling; E, *E2F* targets; F, G2M checkpoint; G, *NOTCH* signaling; H, TGF-β signaling; I, angiogenesis).

The trajectory evolution and immunoregulatory potential of γδ T cells in the TME of LUAD had now been largely elucidated, prompting researchers to investigate whether high infiltration of γδ T cells could be regard as a positive prognostic factor for patients. Therefore, we utilized the CIBERSORTx deconvolution algorithm to quantify the infiltration abundance of γδ T cells in each patient and conducted survival analysis in the TCGA-LUAD cohort. Using the best cut-off value of infiltration abundance, patients were categorized into a high-infiltration group (N=84) and a low-infiltration group (N=419). The findings showed a statistically significant association between γδ T cell infiltration and patient survival, with the high-infiltration group demonstrating better survival outcomes (p=0.011, Figure 4A). These findings suggested that the infiltration of γδ T cells could be a positive guide for the prognostic assessment of LUAD patients.

### Somatic mutation landscape with infiltration level of γδ T cell

Be curious about whether somatic gene mutations in tumor cells could influence γδ T cell infiltration, the somatic mutation landscape was conducted in different infiltration groups using the complete SNV data. After integrating the group data and excluding invalid entries, the Fisher's exact test was conducted to analyze significant differences in gene mutations between the two groups. Genes such as *TNN* (p<0.01) and *DNAH9* (p<0.05) showed higher somatic mutation frequencies in the high-infiltration group, whereas *LPA* (p<0.01), *DNAH8* (p<0.01), *TIAM1* (p<0.05), *ASPM* (p<0.05), and *UNC5D* (p<0.05) exhibited higher mutation frequencies in the low-infiltration abundance group (Figure 4B). Notably, *TNN* gene displayed more missense mutations in the high-infiltration abundance group, while *DNAH9* showed both more missense mutations and multi-hits in this group (Figure 4C). These results suggested significant differences in somatic mutations between patients with varying levels of γδ T cell infiltration, indicating a nuanced association between γδ T cell infiltration and somatic gene mutations.

### High infiltration of γδ T cells is correlated with the downregulation of malignant pathways

Building on the differential analysis of groups with different γδ T cell infiltration abundance levels, we further investigated the potential mechanisms underlying these differences. After obtaining the list of differentially expressed genes using bulk transcriptome data, we ranked them by fold change and performed GSEA functional enrichment analysis to identify the molecular pathways potentially affected by γδ T cell infiltration. Among the top 25 pathways in the GSEA analysis, we observed a marked downregulation of several tumor-associated malignant pathways as γδ T cell infiltration increased. Specifically, the high γδ T cell infiltration abundance group showed a downregulation of the *KRAS* signaling (NES = -1.47, adjusted p = 0.04), *E2F* targets (NES = -1.48, adjusted p = 0.01), G2M checkpoint (NES = -1.6, adjusted p < 0.001), *Notch* signaling (NES = -2.18, adjusted p < 0.001), TGF-β signaling (NES = -2.01, adjusted p < 0.01), and angiogenesis pathways (NES = -1.68, adjusted p = 0.03) (Figures 4D-I, Supplementary File: Table S1). Since *KRAS* is a well-recognized oncogene in the NSCLC, its inhibition could potentially reduce tumor progression. These findings indicate that γδ T cells may inhibit tumor growth and metastasis by modulating various signaling pathways, including those involved in proliferation, immune response, and angiogenesis, thereby exerting anti-tumor effects.

### Patients with higher γδ T cell infiltration abundance derive more advantage from immunotherapy

**Figure 5:**
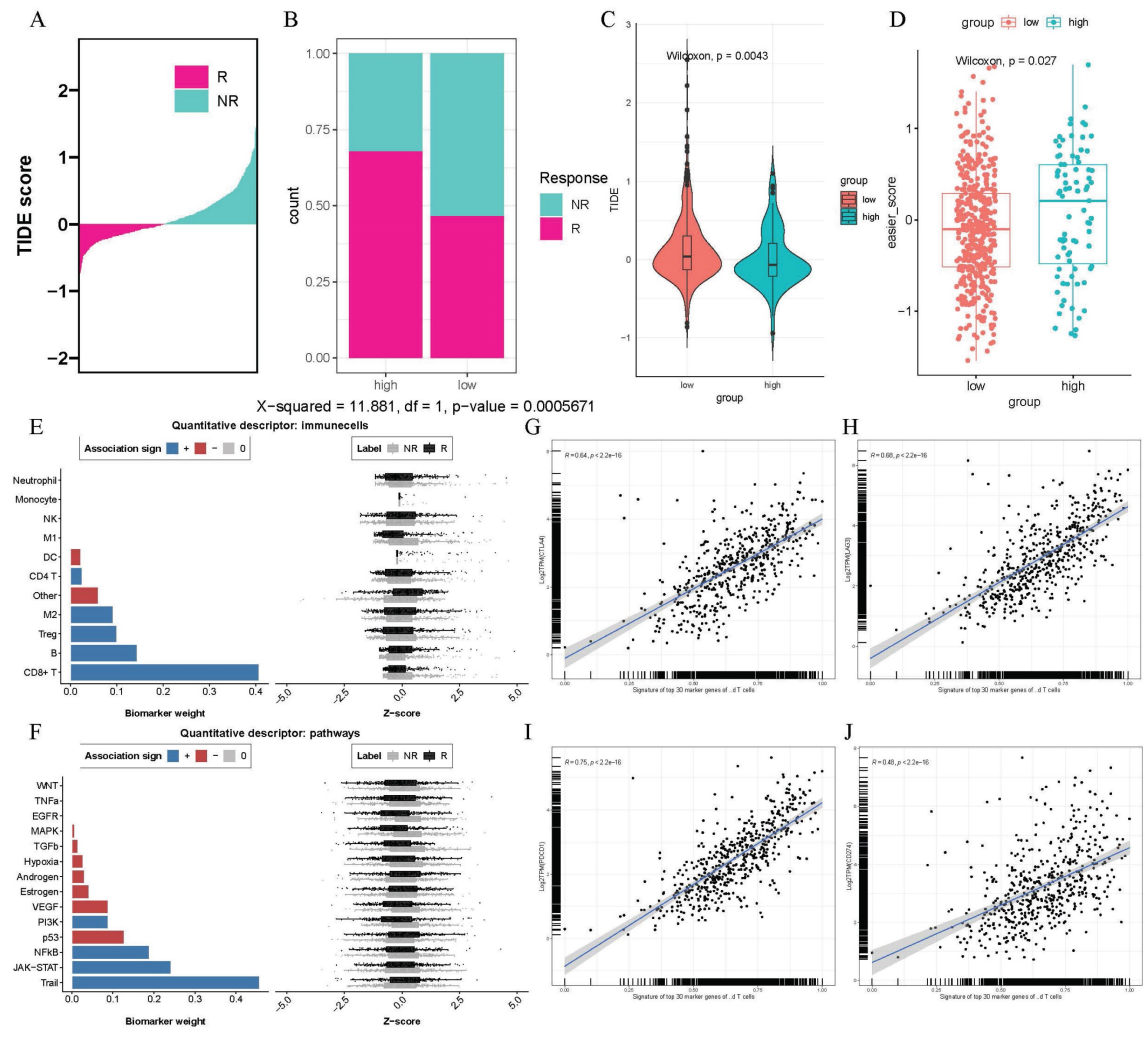
** High γδ T cell infiltration is associated with greater benefits from immunotherapy.** (A-B) Bar chart and stacked plot indicating TIDE data on responsiveness to immunotherapy. (C) Violin plot comparing TIDE scores between high- and low- γδ T cells infiltration abundance groups. (D) Scatter plot of EaSIer scores in high- and low- γδ T cells infiltration abundance groups. The bar chart and Z-score scatter plot revealing the contribution weights to immunotherapy of different immune cells (E) and biological pathways (F). The scatter plot shows a significant positive correlation between the expression levels of the top 30 genes characterizing γδ T cells and the expression levels of *CTLA4* (G), *LAG3* (H), *PDCD1* (I), and C*D273* (J).

Immunotherapy, especially immune checkpoint inhibitor therapy had revolutionized the treatment landscape for lung adenocarcinoma in recent years. Given the immunoregulatory function of γδ T cells, we intended to analyze whether their infiltration could influence the response to immunotherapy. We observed that the high γδ T cell infiltration abundance group had a greater number of responders to immunotherapy according to the TIDE results (p < 0.001, Figures 5A-B). Furthermore, high-infiltration group exhibited lower TIDE scores, suggesting a higher probability of receiving benefits from immunotherapy (p < 0.0043, Figure 5C). Additionally, we observed that patients in the high γδ T cell infiltration group had higher EaSIeR algorithm scores combining TIDE responder result and TMB data, suggesting a more favorable immune microenvironment and a stronger response to immunotherapy (p < 0.027, Figure 5D). In our analysis of immune cells, we found that CD8+ T cells played a crucial role in the γδ T cell-mediated response to immunotherapy (Figure 5E). Notably, the TRAIL pathway showed the strongest positive contribution to the immune response, suggesting it may be a potential pathway for γδ T cell-mediated immunoregulation (Figure 5F). Applying the Seurat package, we identified the top 30 marker genes of γδ T cells (p < 0.05) and converted them into a cell signature gene enrichment score matrix via ssGSEA. We discovered a positive correlation between the expression of γδ T cell signatures and the immune checkpoint genes *CTLA4* (R = 0.64, p < 2.2e-16), *LAG3* (R = 0.68, p < 2.2e-16), *PDCD1* (R = 0.75, p < 2.2e-16), and *CD274* (R = 0.48, p < 2.2e-16) (Figures 5G-J) by Pearson correlation analysis. Therefore, the results mentioned above implied that patients with high γδ T cell infiltration abundance might be better candidates for immunotherapy strategies.

### γδ T cells infiltration abundance can be measured using a radiomics model based on CT imaging

**Figure 6:**
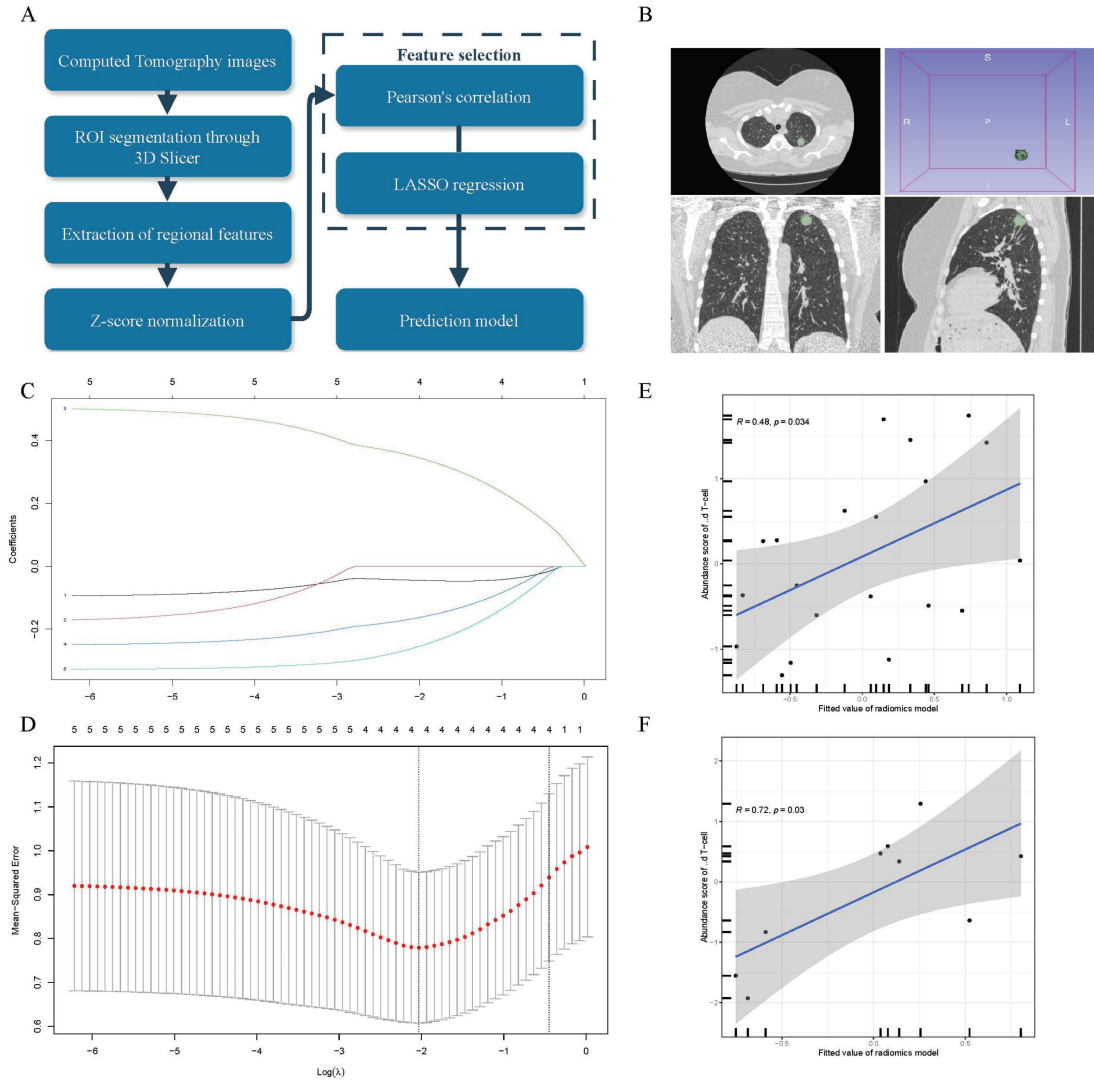
** A LASSO linear regression model was established based on radiomics analysis of CT images, serving as a non-invasive strategy to estimate γδ T cells.** (A) Schematic diagram illustrating the radiomics analysis process. (B) Example of the ROIs (Region of Interest) segmentation in 3D Slicer software. (C) Coefficient distribution of variables in LASSO regression. Each curve represents radiomic features filtered by Pearson correlation. The y-axis indicates their corresponding coefficients in the LASSO regression. The top value represents the number of radiomic features selected through Pearson correlation and included in the LASSO regression model. (D) Parameter tuning plot in Least Absolute Shrinkage and Selection Operator (LASSO) regression. The top value has the same meaning as shown in panel C. Correlation between the abundance of γδ T cells measured by transcriptomics and the fitted values (predicted γδ T cell abundance) from the linear regression radiomic model in the training set (E) and test set (F). The gray area represents the range of 95% confidence intervals (CI).

To further enhance the clinical translational value of this study, we established a non-invasive strategy to assess γδ T cell infiltration levels, as illustrated in Figure 6A. We contained 29 LUAD patients from the TCIA database, and experienced oncologists manually delineated the regions of interest (ROIs) using 3D Slicer. These segmentations were then reviewed and refined by senior clinicians to precisely define the tumor areas and extract relevant feature values (Figure 6B). After extracting 851 features, we used Pearson correlation analysis to identify five features associated with γδ T cell infiltration abundance. Further dimensionality reduction was performed using LASSO regression (Figure 6C-D), resulting in four radiomic features that were selected for constructing the final radiomics linear regression model, detailed in Supplementary File: Table S2. To assess the model's performance, we divided the cohort into a training set (n=20) and a validation set (n=9) using a 2:1 ratio and conducted correlation analysis. We discovered a positive correlation between the γδ T cells infiltration and radiomics scores in both the training set (Figure 6E, R = 0.48, p = 0.034) and the validation set (Figure 6F, R = 0.72, p = 0.03). In conclusion, this study not only explored the function and significance of γδ T cells in the TME of LUAD patients but also established an innovative and non-invasive method for predicting γδ T cell infiltration abundance.

## Discussion

In accordance with the existing reports, our study is the first to comprehensively clarify the role of γδ T cells in the lung adenocarcinoma (LUAD) tumor microenvironment through the integration of multi-omics data. We perform a systematic and multi-layered analysis by integrating single-cell transcription, RNA-sequencing, radiomics, and genomics data. Our research comprehensively uncovered the infiltration of γδ T cells within the TME of LUAD, including their intrinsic lineage evolution, their direct or indirect tumor-killing effects, their impact on immunotherapy, their association with radiomic features, and their cell-to-cell interactions.

The part played by γδ T cells in lung adenocarcinoma remained controversial in previous studies. There were reports suggested that γδ T cells not only served as a favorable prognostic marker in lung cancer but also exhibited stem cell-like characteristics, which might enable them to self-renew within the tumor. Furthermore, it was more likely for the patients with higher γδ T cell infiltration abundance to respond to immunotherapy with pembrolizumab^18^. Clinical trials reported that immunotherapy with autologous γδ T cells have shown to be secure and practicable in NSCLC, and for those patients who were resistant to other treatment, zoledronate-expanded γδ T cells could represent a viable therapeutic option^40^, ^41^. Moreover, studies have shown that immunotherapy of allogeneic γδ T cell can extend the survival time of advanced lung cancer patients^42^. However, Jin C. *et al.* found that γδ T cells could also promote the progression of lung adenocarcinoma through the mediation of commensal microbiota^43^. The potential pro-tumorigenic role of γδ T cells is also a subject that warrants further investigation^44^. Research has demonstrated that γδ T cells in the TME exhibit functional plasticity, engaging in dynamic interactions that toggle between anti- and pro-tumor activities, particularly γδ T cells with the expression of IL-17, which may drive malignant phenotypes in tumors^3^. Given these findings, it is essential to integrate multi-omics data to further clarify the role of γδ T cells in lung adenocarcinoma patients. This study employs a digital assessment strategy based on sc-RNA and bulk transcription data to quantify γδ T cells infiltration abundance in the LUAD microenvironment, revealing that they play an overall positive role in tumor initiation, progression, prognosis, and treatment.

Even without addressing the factors influencing γδ T cell infiltration abundance, our study remains exhaustive and compelling. γδ T cells in the TME can exhibit a high degree of heterogeneity, with significant functional differences among different subtypes^45^. Our research identified an intrinsic lineage evolution of γδ T cells in lung adenocarcinoma TME, where these cells tend to develop and mature towards the expression of *GZMK*,* XCL1,* and *XCL2*. *GZMK* (Granzyme K) as a serine protease of the granzyme family, is primarily expressed by cytotoxic T cells and natural killer (NK) cells, which is characterized by its cytotoxic properties^46^. Therefore, based on the expression of *GZMK* and its developmental trajectory within the γδ T cell population, we artificially defined them into two distinct subgroups: *GZMK-* and *GZMK+*. The *GZMK+* subset represents a more mature cell state with a tendency towards cytotoxic functionality. *XCL1* (lymphotactin1) and *XCL2* (lymphotactin2) both belong to the XC subfamily of chemokines, and they have an impact on regulating immune cell migration, promoting maturation and activation, thus boosting the immune system's response. Notably, NK cells are also capable of expressing various chemokines, including *XCL1*^47^. Our study discovered that γδ T cells may recruit additional immune cells through the secretion of chemokines, thereby indirectly modulating immune function. Dividing γδ T cells into these two differentiated cellular states facilitates a more precise understanding of their critical role in LUAD. IFN-γ is a key regulatory factor in the tumor microenvironment which exerts a broad spectrum of influence and could be considered as the "master regulator" of the immune microenvironment^48^. We observed that γδ T cells transmit signals through the IFN-II (IFN-γ) pathway to macrophages and monocytes. Notably, CD8 Trms (tissue-resident memory T cells) also exhibit strong signaling interactions with macrophages and monocytes via this pathway. We hypothesize that there may be a synergistic relationship between CD8 Trms and γδ T cells. Additionally, it was observed that γδ T cells have cell-to-cell signaling with professional antigen-presenting cells (APCs, e.g., pDCs, monocytes, and macrophages) via MHC-II molecules, thereby enhancing their antigen-presenting capabilities and regulating immune function. *CCL5* is a chemokine reported to recruit more immune cells^49^, while vascular endothelial *ACKR1* facilitates the translocation of chemokines across blood vessels^50^. We found that γδ T cells significantly interact with endothelial cells via the *CCL-ACKR1* signaling pathway. Endothelial cells primarily constitute the vascular walls in the TME, suggesting that γδ T cells may remodel the tumor microenvironment by recruiting immune cells and altering the permeability of endothelial cells. Using a digital deconvolution algorithm, we predicted that patients with higher γδ T cell infiltration abundance have better prognoses. Additionally, through GSEA functional enrichment analysis, we discovered the association between the increasing γδ T cell infiltration abundance and the downregulation of pathways such as *KRAS*,* E2F*, G2M checkpoint, *Notch*, TGF-β, and angiogenesis which are related to malignant tumor phenotypes^51^, ^52^. In conclusion, these findings highlight the critical role of γδ T cells in the LUAD microenvironment.

Immunotherapy has gradually become the mainstream treatment for LUAD in recent years, prompting researchers to further explore the potential value of γδ T cells in this context. We found that patients in the high infiltration group of γδ T cells exhibited better responses to immunotherapy. The treatment process was found to be strongly correlated with the TRAIL and JAK-STAT pathways, with the TRAIL pathway known to induce cell death^53^, ^54^, which may represent a potential mechanism of γδ T cells to exert biological functions. Interestingly, the JAK-STAT pathway has been reported to be activated by IFN-γ^55^, ^56^, which aligns with our findings in CellChat. Moreover, the infiltration abundance was positively correlated with common immunotherapy targets such as *CTLA4*, *LAG3*, *PDCD1*, and *CD274*, indicating that these patients are more suitable for immunotherapy and have significant clinical value. Through somatic mutation analysis, we discovered that a high abundance of γδ T cell infiltration in LUAD is associated with the *TNN* and *DNAH8* genes mutations, suggesting that these mutations may contribute to the increased infiltration of γδ T cells. Reports on the *TNN* gene primarily focus on non-oncological diseases^57^, while studies on the *DNAH8* gene mainly center on male infertility^58^. Our research provides new insight into the study of these two genes.

Radiomics provides a convenient, cost-effective, reproducible, and non-invasive strategy for the clinical assessment of biological characteristics. In our research, we aimed to establish an innovative non-invasive method for evaluating the infiltration abundance of γδ T cells to demonstrate its practical value. Despite the limitation of sample number, we ensured the accuracy of our data sources as much as possible. We employed LASSO to introduce an L1 regularization term, which helped to avoid multicollinearity and improve the model's predictive capability. Our research indicates that a radiomics model established based on CT imaging features can predict the infiltration abundance of γδ T cells, meanwhile its correlation was explored in both the training and validation datasets. This represents a bold innovative attempt, and we hope it will lay the groundwork for further research.

It bears mentioning that this research is an exploratory investigation based on multi-omics data which has certain limitations. Even though we employed a comprehensive approach, the sample size of sc-RNA data may limit the generalizability of our findings. Similarly, the scarcity of imaging data presents challenges to the stability of our model. We plan to collect more sample to validate the universality of our model. Furthermore, the clinical significance and translational value of γδ T cells require confirmation from multi-center large sample cohorts. Owing to the limited abundance of γδ T cells and the difficulties of establishing animal models^59^, we anticipate difficulties and challenges in elucidating their molecular mechanisms. However, despite the need for further research validation, we are convinced that the assessment results of γδ T cells are reliable based on our relatively comprehensive research process. By investigating the immunomodulatory and antitumor effects of γδ T cells in the LUAD microenvironment, this study is expected to deepen our understanding of the unique mechanisms of γδ T cells.

## Supplementary Material

Supplementary figures.

Supplementary table 1.

Supplementary table 2.

## Abbreviations

LUAD: lung adenocarcinoma
IFN: interferon
TNF: tumor necrosis factor
TCGA: the cancer genome atlas program
TCIA: the cancer imaging archive
GEO: gene expression omnibus
LASSO: least absolute shrinkage and selection operator
MHC: major histocompatibility complex
NSCLC: non-small cell lung cancer
TME: tumor microenvironment
GSEA: gene set enrichment analysis
SNV: single nucleotide variant
TMB: tumor mutation burden
ICIs: immune checkpoint inhibitors
ROI: region of interest
CT: computed tomography

## References

[1] Sebestyen Z, Prinz I, Dechanet-Merville J, Silva-Santos B, Kuball J (2020). Translating gammadelta (gammadelta) T cells and their receptors into cancer cell therapies. Nat Rev Drug Discov.

[2] Wu YL, Ding YP, Tanaka Y, Shen LW, Wei CH, Minato N (2014). gammadelta T cells and their potential for immunotherapy. Int J Biol Sci.

[3] Mensurado S, Blanco-Dominguez R, Silva-Santos B (2023). The emerging roles of gammadelta T cells in cancer immunotherapy. Nat Rev Clin Oncol.

[4] Hayday A, Dechanet-Merville J, Rossjohn J, Silva-Santos B (2024). Cancer immunotherapy by gammadelta T cells. Science.

[5] Reis BS, Darcy PW, Khan IZ, Moon CS, Kornberg AE, Schneider VS (2022). TCR-Vgammadelta usage distinguishes protumor from antitumor intestinal gammadelta T cell subsets. Science.

[6] Rei M, Goncalves-Sousa N, Lanca T, Thompson RG, Mensurado S, Balkwill FR (2014). Murine CD27(-) Vgamma6(+) gammadelta T cells producing IL-17A promote ovarian cancer growth via mobilization of protumor small peritoneal macrophages. Proc Natl Acad Sci U S A.

[7] Leiter A, Veluswamy RR, Wisnivesky JP (2023). The global burden of lung cancer: current status and future trends. Nat Rev Clin Oncol.

[8] Bray F, Laversanne M, Sung H, Ferlay J, Siegel RL, Soerjomataram I (2024). Global cancer statistics 2022: GLOBOCAN estimates of incidence and mortality worldwide for 36 cancers in 185 countries. CA Cancer J Clin.

[9] Siegel RL, Giaquinto AN, Jemal A (2024). Cancer statistics, 2024. CA Cancer J Clin.

[10] Cronin KA, Lake AJ, Scott S, Sherman RL, Noone AM, Howlader N (2018). Annual Report to the Nation on the Status of Cancer, part I: National cancer statistics. Cancer.

[11] Herbst RS, Morgensztern D, Boshoff C (2018). The biology and management of non-small cell lung cancer. Nature.

[12] Skoulidis F, Heymach JV (2019). Co-occurring genomic alterations in non-small-cell lung cancer biology and therapy. Nat Rev Cancer.

[13] de Sousa VML, Carvalho L (2018). Heterogeneity in Lung Cancer. Pathobiology.

[14] Yu H, Zhang W, Xu XR, Chen S (2023). Drug resistance related genes in lung adenocarcinoma predict patient prognosis and influence the tumor microenvironment. Sci Rep.

[15] Wang G, Shi C, He L, Li Y, Song W, Chen Z (2024). Identification of the tumor metastasis-related tumor subgroups overexpressed NENF in triple-negative breast cancer by single-cell transcriptomics. Cancer Cell Int.

[16] Liu C, Li X, Huang Q, Zhang M, Lei T, Wang F (2023). Single-cell RNA-sequencing reveals radiochemotherapy-induced innate immune activation and MHC-II upregulation in cervical cancer. Signal Transduct Target Ther.

[17] Chen M, Copley SJ, Viola P, Lu H, Aboagye EO (2023). Radiomics and artificial intelligence for precision medicine in lung cancer treatment. Semin Cancer Biol.

[18] Wu Y, Biswas D, Usaite I, Angelova M, Boeing S, Karasaki T (2022). A local human Vdelta1 T cell population is associated with survival in nonsmall-cell lung cancer. Nat Cancer.

[19] Mitsuhashi A, Koyama K, Ogino H, Afroj T, Nguyen NT, Yoneda H (2023). Identification of fibrocyte cluster in tumors reveals the role in antitumor immunity by PD-L1 blockade. Cell Rep.

[20] Colaprico A, Silva TC, Olsen C, Garofano L, Cava C, Garolini D (2016). TCGAbiolinks: an R/Bioconductor package for integrative analysis of TCGA data. Nucleic Acids Res.

[21] Clark K, Vendt B, Smith K, Freymann J, Kirby J, Koppel P (2013). The Cancer Imaging Archive (TCIA): maintaining and operating a public information repository. J Digit Imaging.

[22] McGinnis CS, Murrow LM, Gartner ZJ (2019). DoubletFinder: Doublet Detection in Single-Cell RNA Sequencing Data Using Artificial Nearest Neighbors. Cell Syst.

[23] Korsunsky I, Millard N, Fan J, Slowikowski K, Zhang F, Wei K (2019). Fast, sensitive and accurate integration of single-cell data with Harmony. Nat Methods.

[24] Hafemeister C, Satija R (2019). Normalization and variance stabilization of single-cell RNA-seq data using regularized negative binomial regression. Genome Biol.

[25] Zappia L, Oshlack A (2018). Clustering trees: a visualization for evaluating clusterings at multiple resolutions. Gigascience.

[26] Hu C, Li T, Xu Y, Zhang X, Li F, Bai J (2023). CellMarker 2.0: an updated database of manually curated cell markers in human/mouse and web tools based on scRNA-seq data. Nucleic Acids Res.

[27] Trapnell C, Cacchiarelli D, Grimsby J, Pokharel P, Li S, Morse M (2014). The dynamics and regulators of cell fate decisions are revealed by pseudotemporal ordering of single cells. Nat Biotechnol.

[28] Jin S, Guerrero-Juarez CF, Zhang L, Chang I, Ramos R, Kuan CH (2021). Inference and analysis of cell-cell communication using CellChat. Nat Commun.

[29] Newman AM, Steen CB, Liu CL, Gentles AJ, Chaudhuri AA, Scherer F (2019). Determining cell type abundance and expression from bulk tissues with digital cytometry. Nat Biotechnol.

[30] Ritchie ME, Phipson B, Wu D, Hu Y, Law CW, Shi W (2015). limma powers differential expression analyses for RNA-sequencing and microarray studies. Nucleic Acids Res.

[31] Subramanian A, Tamayo P, Mootha VK, Mukherjee S, Ebert BL, Gillette MA (2005). Gene set enrichment analysis: a knowledge-based approach for interpreting genome-wide expression profiles. Proc Natl Acad Sci U S A.

[32] Wu T, Hu E, Xu S, Chen M, Guo P, Dai Z (2021). clusterProfiler 4.0: A universal enrichment tool for interpreting omics data. Innovation (Camb).

[33] Mayakonda A, Lin DC, Assenov Y, Plass C, Koeffler HP (2018). Maftools: efficient and comprehensive analysis of somatic variants in cancer. Genome Res.

[34] Fu J, Li K, Zhang W, Wan C, Zhang J, Jiang P (2020). Large-scale public data reuse to model immunotherapy response and resistance. Genome Med.

[35] Lapuente-Santana O, van Genderen M, Hilbers PAJ, Finotello F, Eduati F (2021). Interpretable systems biomarkers predict response to immune-checkpoint inhibitors. Patterns (N Y).

[36] Hanzelmann S, Castelo R, Guinney J (2013). GSVA: gene set variation analysis for microarray and RNA-seq data. BMC Bioinformatics.

[37] Fedorov A, Beichel R, Kalpathy-Cramer J, Finet J, Fillion-Robin JC, Pujol S (2012). 3D Slicer as an image computing platform for the Quantitative Imaging Network. Magn Reson Imaging.

[38] Zwanenburg A, Vallieres M, Abdalah MA, Aerts H, Andrearczyk V, Apte A (2020). The Image Biomarker Standardization Initiative: Standardized Quantitative Radiomics for High-Throughput Image-based Phenotyping. Radiology.

[39] Friedman J, Hastie T, Tibshirani R (2010). Regularization Paths for Generalized Linear Models via Coordinate Descent. J Stat Softw.

[40] Nakajima J, Murakawa T, Fukami T, Goto S, Kaneko T, Yoshida Y (2010). A phase I study of adoptive immunotherapy for recurrent non-small-cell lung cancer patients with autologous gammadelta T cells. Eur J Cardiothorac Surg.

[41] Sakamoto M, Nakajima J, Murakawa T, Fukami T, Yoshida Y, Murayama T (2011). Adoptive immunotherapy for advanced non-small cell lung cancer using zoledronate-expanded gammadeltaTcells: a phase I clinical study. J Immunother.

[42] Xu Y, Xiang Z, Alnaggar M, Kouakanou L, Li J, He J (2021). Allogeneic Vgamma9Vdelta2 T-cell immunotherapy exhibits promising clinical safety and prolongs the survival of patients with late-stage lung or liver cancer. Cell Mol Immunol.

[43] Jin C, Lagoudas GK, Zhao C, Bullman S, Bhutkar A, Hu B (2019). Commensal Microbiota Promote Lung Cancer Development via gammadelta T Cells. Cell.

[44] Harmon C, Zaborowski A, Moore H, St Louis P, Slattery K, Duquette D (2023). gammadelta T cell dichotomy with opposing cytotoxic and wound healing functions in human solid tumors. Nat Cancer.

[45] Yan W, Dunmall LSC, Lemoine NR, Wang Y, Wang Y, Wang P (2023). The capability of heterogeneous gammadelta T cells in cancer treatment. Front Immunol.

[46] Bouwman AC, van Daalen KR, Crnko S, Ten Broeke T, Bovenschen N (2021). Intracellular and Extracellular Roles of Granzyme K. Front Immunol.

[47] Lei Y, Takahama Y (2012). XCL1 and XCR1 in the immune system. Microbes Infect.

[48] Hoekstra ME, Slagter M, Urbanus J, Toebes M, Slingerland N, de Rink I (2024). Distinct spatiotemporal dynamics of CD8(+) T cell-derived cytokines in the tumor microenvironment. Cancer Cell.

[49] Zhang XF, Zhang XL, Wang YJ, Fang Y, Li ML, Liu XY (2023). The regulatory network of the chemokine CCL5 in colorectal cancer. Ann Med.

[50] Mempel TR, Lill JK, Altenburger LM (2024). How chemokines organize the tumour microenvironment. Nat Rev Cancer.

[51] Li Y, Wu J, Tian Y, Zhu Q, Ge Y, Yu H (2022). MED1 Downregulation Contributes to TGFbeta-Induced Metastasis by Inhibiting SMAD2 Ubiquitination Degradation in Cutaneous Melanoma. J Invest Dermatol.

[52] Tian Y, Yu Y, Hou LK, Chi JR, Mao JF, Xia L (2016). Serum deprivation response inhibits breast cancer progression by blocking transforming growth factor-beta signaling. Cancer Sci.

[53] Hartwig T, Montinaro A, von Karstedt S, Sevko A, Surinova S, Chakravarthy A (2017). The TRAIL-Induced Cancer Secretome Promotes a Tumor-Supportive Immune Microenvironment via CCR2. Mol Cell.

[54] Johnstone RW, Frew AJ, Smyth MJ (2008). The TRAIL apoptotic pathway in cancer onset, progression and therapy. Nat Rev Cancer.

[55] Philips RL, Wang Y, Cheon H, Kanno Y, Gadina M, Sartorelli V (2022). The JAK-STAT pathway at 30: Much learned, much more to do. Cell.

[56] Tehrani SS, Mikulski P, Abdul-Zani I, Mata JF, Siwek W, Jansen LE (2023). STAT1 is required to establish but not maintain interferon-gamma-induced transcriptional memory. EMBO J.

[57] Tadros HJ, Life CS, Garcia G, Pirozzi E, Jones EG, Datta S (2020). Meta-analysis of cardiomyopathy-associated variants in troponin genes identifies loci and intragenic hot spots that are associated with worse clinical outcomes. J Mol Cell Cardiol.

[58] Liu C, Miyata H, Gao Y, Sha Y, Tang S, Xu Z (2020). Bi-allelic DNAH8 Variants Lead to Multiple Morphological Abnormalities of the Sperm Flagella and Primary Male Infertility. Am J Hum Genet.

[59] Papadopoulou M, Sanchez Sanchez G, Vermijlen D (2020). Innate and adaptive gammadelta T cells: How, when, and why. Immunol Rev.

