# Supplementary material for: Integrating of Radiomics and Single-cell Transcriptomics Uncovers the Crucial Role of γδ T Cells in Lung Adenocarcinoma
Source: J Cancer. 2025 Jan 6;16(4):1167–80. doi: 10.7150/jca.105586 (PMC11786044; doi:10.7150/jca.105586)
Supplement: Supplementary file 1 — Supplementary figures. [file jcav16p1167s1.pdf]

Supplementary Figures

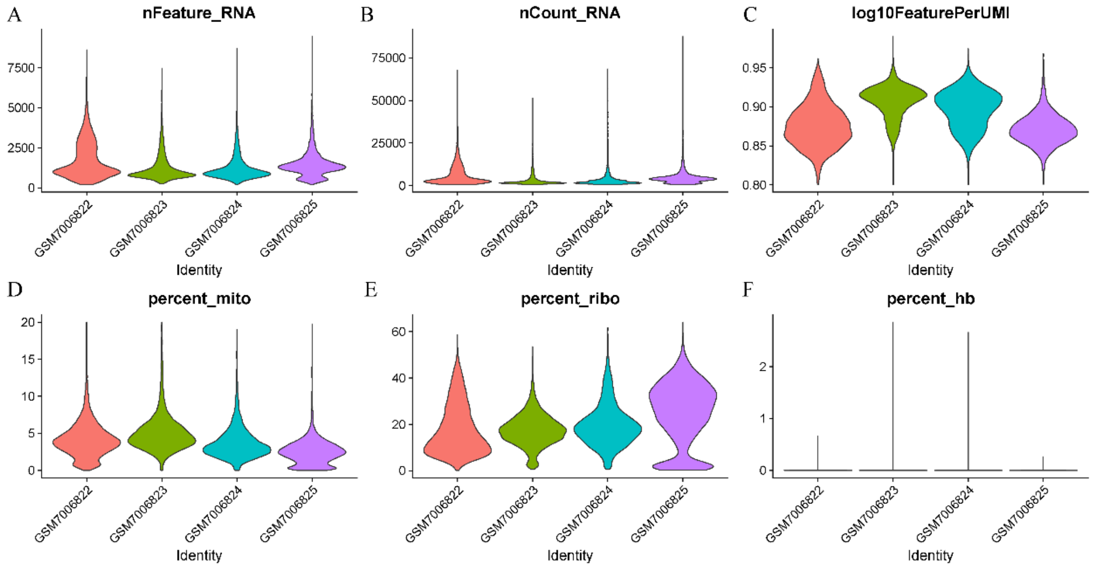

Supplementary Figure 1 The schematic of the quality control standards used for the single-cell data in the study. (A) Genes detected per cell was set to ≥ 200. (B) Total number of RNA reads detected per cell was set to ≥ 500. (C) Log10 of the ratio of the number of gene features to the number of UMIs for each cell was set to ≥ 0.8. (D) The proportion of reads to mitochondrial genes was set to ≤ 20%. (E) No threshold was set for the proportion of reads to ribosomal genes. (F) The proportion of reads to hemoglobin genes was set to ≤ 3%.

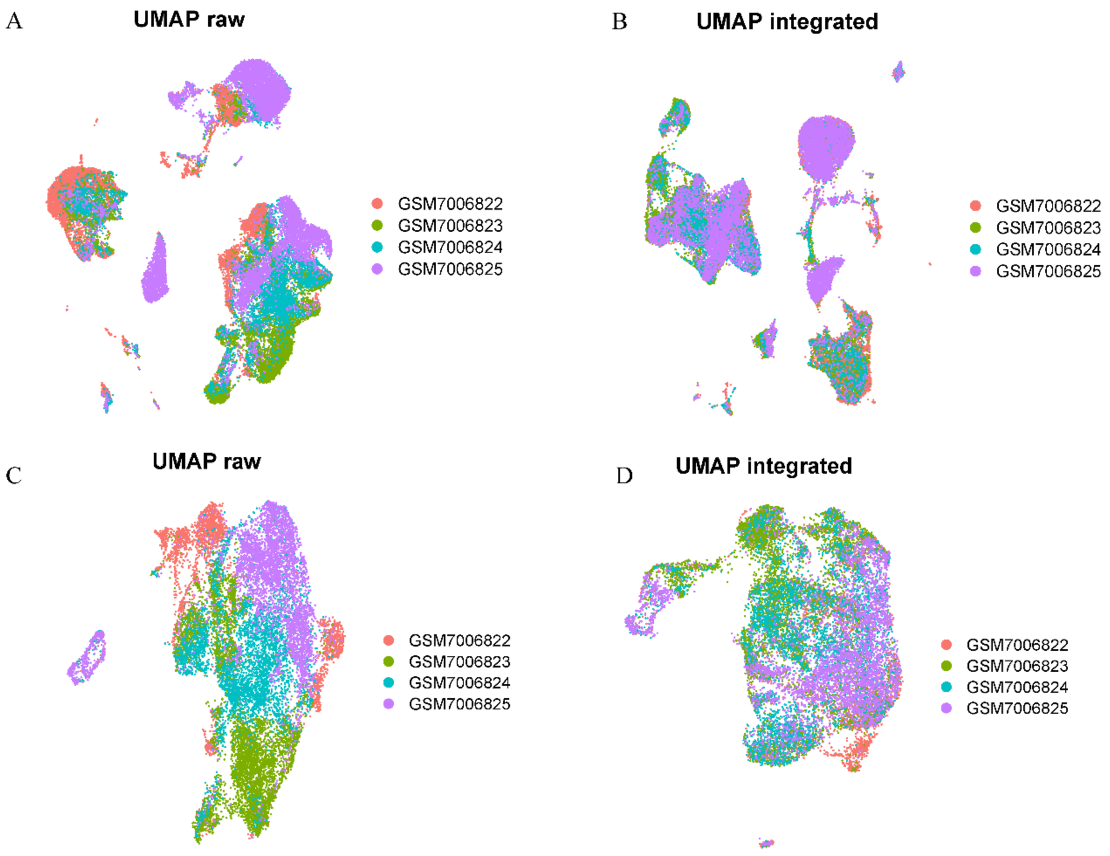

Supplementary Figure 2 UMAP before and after the removal of batch effect. (A) UMAP of the overall sc-RNA data before the removal of batch effect. (B) UMAP of the overall sc-RNA data after the removal of batch effect. (C) UMAP of the extracted T cells sc-RNA data before the removal of batch effect. (D) UMAP of the extracted T cells sc-RNA data after the removal of batch effect
